# Bursectomy for advanced gastric cancer: an update meta-analysis

**DOI:** 10.1186/s12957-018-1354-1

**Published:** 2018-03-27

**Authors:** Run-Cong Nie, Shu-Qiang Yuan, Shi Chen, Shu-Mei Yan, Yong-Ming Chen, Xiao-Jiang Chen, Guo-Ming Chen, Zhi-Wei Zhou, Ying-Bo Chen, Yuan-Fang Li

**Affiliations:** 1 Department of Gastric Surgery, Sun Yat-sen University Cancer Center; State Key Laboratory of Oncology in South China; Collaborative Innovation Center for Cancer Medicine, 651 E Dongfeng Road, Guangzhou, Guangdong 510060 China; 2 Department of Gastric Surgery, The 6th Affiliated Hospital, Sun Yat-sen University, Guangzhou, China; 3 Department of Pathology, Sun Yat-sen University Cancer Center; State Key Laboratory of Oncology in South China; Collaborative Innovation Center for Cancer Medicine, 651 E Dongfeng Road, Guangzhou, Guangdong 510060 China

**Keywords:** Bursectomy, Gastric cancer, Survival, Meta-analysis

## Abstract

**Background:**

The present meta-analysis was to explore the surgical and oncological outcomes of bursectomy for advanced gastric cancer (AGC).

**Methods:**

Relevant studies that evaluated the role of bursectomy for AGC were comprehensively examined to perform a meta-analysis. The primary outcomes were overall survival (OS) and disease-free survival (DFS). The secondary outcomes were the number of harvested lymph nodes (LNs), operation time, operative bleeding, hospital stay, postoperative complication and mortality.

**Results:**

A total of seven studies comprising 2633 cases (1176 cases in the bursectomy group and 1457 cases in the non-bursectomy group) were finally included. There was no significant difference in OS (HR 0.95, *P* = 0.647) and DFS (HR 0.99, *P* = 0.936) between the two groups. Even for patients with serosa-penetrating tumours, OS was comparable between the two groups (HR 0.87, *P* = 0.356). The operation time of the bursectomy group was longer (weighted mean difference, WMD 32.76 min, *P* = 0.002). No significant difference was found between the two groups in terms of the number of dissected LNs (WMD 5.86, *P* = 0.157), operative bleeding (WMD 66.99 ml, *P* = 0.192) and hospital stay (WMD − 0.15 days, *P* = 0.766). The overall postoperative complication (relative risk, RR 1.08, *P* = 0.421) and mortality (RR 0.44, *P* = 0.195) were similar between two groups.

**Conclusions:**

This meta-analysis indicated that bursectomy is time-consuming without increasing the number of harvested LNs. Although bursectomy can be safely performed without increasing complications and mortality, it does not prolong the OS and DFS of AGC patients, including patients with serosa-penetrating tumours. Therefore, bursectomy should not be recommended as a standard procedure for AGC.

**Electronic supplementary material:**

The online version of this article (10.1186/s12957-018-1354-1) contains supplementary material, which is available to authorized users.

## Background

Gastric carcinoma is the third leading cause of cancer-related deaths worldwide, causing estimated 723,100 deaths in 2012 [1]. Gastrectomy with D2 lymphadenectomy is recommended as the standard strategy for curable gastric cancer, especially in eastern Asia [2, 3]. However, approximately 30–40% advanced gastric cancer (AGC) patients suffer from recurrence after radical surgery and subsequent adjuvant chemotherapy [4, 5]. Among the patterns of recurrence, peritoneal metastasis is the most common.

Bursectomy is a procedure of dissecting the peritoneal lining covering the pancreas and the anterior plane of the transverse mesocolon. Since the 1960s in Japan, bursectomy has been recommended as a part of radical gastrectomy for AGC, especially for tumours invading the serosa of the posterior gastric wall [6]. Theoretically, bursectomy can promote complete dissection of subpyloric lymph nodes (LNs) and inclusion of potential lesser peritoneal sac micrometastases within the resection. Imamura et al. reported that the overall postoperative morbidity and mortality were comparable between the bursectomy group and non-bursectomy group, indicating that it was safe to perform D2 gastrectomy with bursectomy [7]. In 2015, Hirao et al. reported the results of a final analysis of a multicenter randomised controlled trial (RCT) [8], suggesting that bursectomy should not be abandoned because of its survival benefit shown in the Cox multivariate analysis (HR 0.58, *P* = 0.034). However, patients included in this study did not permit adjuvant chemotherapy, which attributed to early closure of the trial. At the 2017 American Society of Clinical Oncology Gastrointestinal Cancers Symposium (ASCO-GI), Masanori et al. [9] reported the primary results of a phase III trial evaluating bursectomy for patients with T3/4 gastric cancer (JCOG1001). The results demonstrated that the 3-year OS was similar between the bursectomy group and non-bursectomy group (86.0 vs. 83.3%). Therefore, the role of bursectomy for AGC remained controversial.

A meta-analysis by Shen et al. investigated the role of D2 gastrectomy with bursectomy for AGC [10]. However, one of the included studies in that meta-analysis explored the role of omentectomy for AGC [11]. Only 113 patients had undergone bursectomy in the omentectomy group (180 patients). Therefore, the design of this meta-analysis was not rigorous, and the results of this meta-analysis were confounded and questionable. Our purpose was to perform a rigorous updated meta-analysis to investigate the surgical and oncological outcomes of D2 gastrectomy plus bursectomy.

## Methods

### Search strategy and study selection

Two authors (RC, Nie and SQ, Yuan) searched electronic databases including Medline, Web of Science and EMBASE systematically to retrieve the relevant studies on April 7th, 2017. The following terms in [Title/Abstract] were used: “omental bursa resection”, “bursectomy”, “gastric cancer”, “gastric carcinoma”, “gastric neoplasm”, “stomach cancer” and “stomach neoplasm”. We also screened the reference lists of the retrieved studies manually to supplement the results. We searched the conference reports presented at annual meetings of the American Society of Clinical Oncology (ASCO) and European Society for Medical Oncology (ESMO) for supplementary studies.

Studies were included if they evaluated the survival outcome or surgical outcome of bursectomy in gastric cancer patients. Studies were excluded if they were animal studies, case reports, letters, comments, reviews, meta-analysis and studies published in a language other than English. If multiple articles reported overlapping data from the same patient cohort, the most recent study/studies with the most complete data was/were included.

### Data extraction

Data on the characteristics, surgical outcomes and survival outcomes of all included studies were independently extracted by two authors (RC, Nie and SQ, Yuan). The primary outcomes were overall survival (OS) and disease-free survival (DFS). The secondary outcomes were the number of harvested LNs, operation time, operative bleeding, hospital stay, postoperative complication and mortality. The following postoperative complications were extracted: pancreatic fistula, abdominal infection, ileus, haemorrhage and pneumonia. Two senior authors (YF, Li and YB, Chen) resolved any disagreements in data extraction.

### Quality assessment

The levels of evidence of included studies were rated according to criteria by the Centre for Evidence-Based Medicine in Oxford, UK [12]. The quality of all the included studies was independently assessed by two authors (S, Chen and XJ, Chen). The quality of retrospective studies was evaluated using the Newcastle–Ottawa Scale (NOS) [13] with a scale from 0 to 9, and the Cochrane risk of bias tool [14] was used to assess the quality of RCTs. RCTs and retrospective studies with a score higher than five were considered high-quality studies in this analysis.

### Statistical analysis

Weighted mean difference (WMD) and relative risk (RR) were used to analyse continuous and dichotomous data, respectively. Hazard ratios (HRs) were used to assess the survival effect of bursectomy. All results were presented with 95% confidence intervals (CIs). If the included studies presented continuous data as median and range, the results were transformed to mean and standard error as illustrated by Hozo et al. [15]. For studies that did not report HRs with 95% CIs, we calculated the HRs from survival curves according to the method described by Parmar et al. [16]. All the statistical analyses were performed with the *χ*^2^ test using *I*^2^ statistic to assess heterogeneity, with the level of significance set at 10%. The random-effects model was used to pool the data because of different levels of evidence of included studies. We also used Begg’s funnel plot to assess the publication bias. STATA/SE 12.0 (StataCorp LP, College Station, TX) was used to analyse all the data in this meta-analysis.

## Results

### Characteristics of included studies

**Fig. 1:**
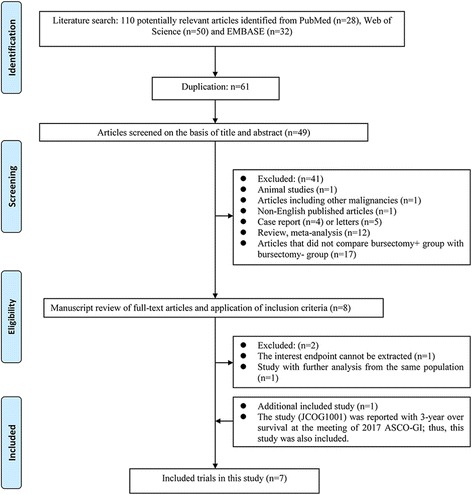
Flowchart of the eligible studies

A total of 110 studies met the initial criteria, and we identified seven studies for final analysis (Fig. 1). The characteristics of included studies are shown in Table 1. The final included studies [7–9, 17–20] comprised 2633 cases (1176 cases in the bursectomy group and 1457 cases in the non-bursectomy group). Among the included studies, there were two RCTs [8, 9] and four retrospective studies [17–20]. The study by Imamura et al. [7] and the study by Hirao et al. [8] were on the same patient cohort. The former reported the surgical outcomes, while the latter reported the long-term survival outcomes. Masanori et al. [9] reported the primary results of JCOG1001 at the 2017 ASCO-GI meeting. All the included studies were considered as high quality.

**Table 1:** Characteristics of the included studies

Author, year	Evidence level	Country	Study period	Study design	TNM stage	Patients		Full text/abstract	Median follow-up (months)	Quality scores	Outcomes
						Bursectomy (+)	Bursectomy (−)				
Imamura 2011, [7]^a^	2b	Japan	2002–2007	RCT	cT2–3	104	106	Full text	NA	RCT	3,4,5,7,8
Eom, 2013 [17]	3b	Korea	2001–2006	R	cT3–4	107	363	Full text	60	7	1,2,3,4,6,7,8
Kochi,2014 [18]	3b	Japan	2004–2010	R	cIA–IIIC	121	133	Full text	60	7	1,2,3,4,5,6,7,8
Kung, 2014 [19]	3b	Sweden	2006–2012	R	All stages	83	8	Full text	NA	5	7^b^
Hirao, 2015 [8]^a^	2b	Japan	2002–2007	RCT	cT2–3	104	106	Full text	80	RCT	1,2
Zhang,2015 [20]	3b	China	2012–2013	R	pT2–4	159	247	Full text	20	6	1,3,4,5,6,7,8
Masanori, 2017 [9]^c^	1b	Japan	2010–2015	RCT	cT3–4	602	602	Abstract	NA	RCT	1,7,8

*R* retrospective study, *RCT* randomised controlled trial, *NA* not available, *1* overall survival, *2* disease-free survival, *3* harvested lymph node, *4* operating time, *5* blood loss, *6* hospital stay, *7* operative morbidity, *8* operative mortality

^a^The study by Imamura et al. and the study by Hirao et al. were on the same patient cohort. The former reported surgical outcomes, while the latter reported long-term survival outcomes

^b^This study aimed to explore the risk of postoperative pancreatic fistula after D2 gastrectomy, including bursectomy, for gastric cancer

^c^This study reports the primary results of a phase III trial evaluating bursectomy for patients with subserosal/serosal gastric cancer (JCOG1001), which was delivered in the form of an oral presentation at the 2017 American Society of Clinical Oncology Gastrointestinal Cancers Symposium (ASCO-GI)

### Primary outcomes

**Fig. 2:**
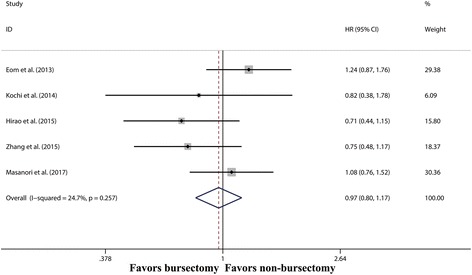
Forest plot and meta-analysis of overall survival

Five studies [8, 9, 17, 18, 20] comprising 2544 patients reported OS without heterogeneity (*I*^2^ = 24.7%, *P* = 0.257). In this study, the random-effects model was used to pool the data because of different levels of evidence of included studies, which meant the potential heterogeneity between included studies. The pooled HR for OS was 0.95 (95% CI 0.76–1.19, *P* = 0.647) (Fig. 2), indicating no survival benefit for bursectomy.

**Fig. 3:**
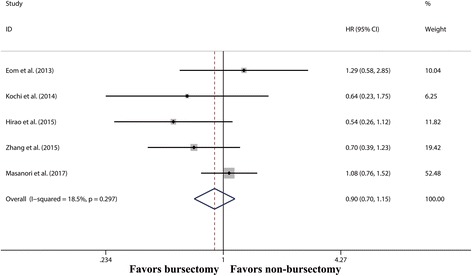
Forest plot and meta-analysis of overall survival in patients with serosa-penetrating tumours

Five studies [8, 9, 17, 18, 20] reported OS of gastric cancer patients with serosa-penetrating tumours, and there was no significant difference in OS between the bursectomy group and non-bursectomy group (HR 0.87, 95% CI 0.64–1.17, *P* = 0.356) (Fig. 3).

**Fig. 4:**
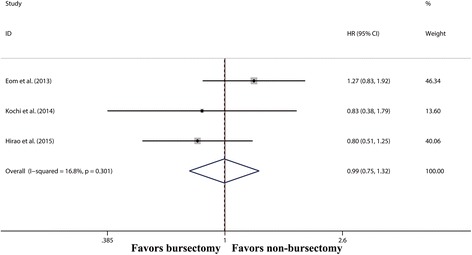
Forest plot and meta-analysis of disease-free survival

Three studies [8, 17, 18] reported DFS between two groups (Fig. 4). The pooled analysis showed that DFS was similar between the bursectomy group and non-bursectomy group (HR 0.99, 95% CI 0.72–1.36, *P* = 0.936).

### Secondary outcomes

**Fig. 5:**
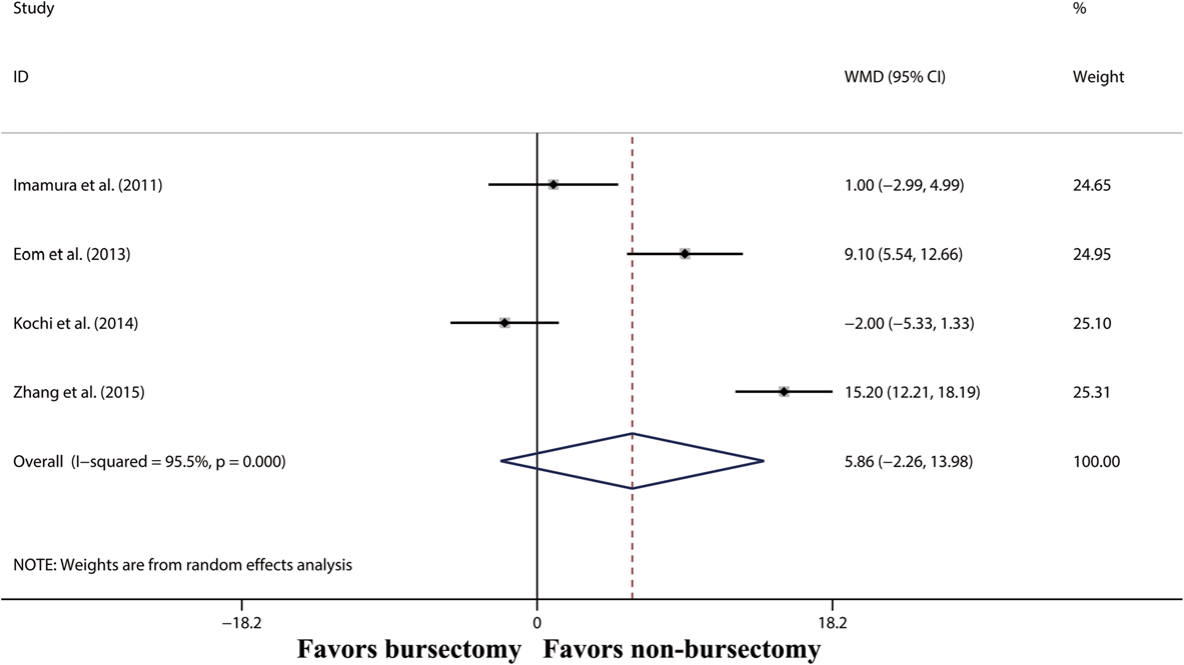
Forest plot and meta-analysis of harvested lymph nodes

As shown in Fig. 5, the bursectomy group did not harvest more LNs than the non-bursectomy group (WMD 5.86, 95% CI − 2.26–13.98, *P* = 0.157). Moreover, compared with the non-bursectomy group, the operation time of the bursectomy group was longer (WMD 32.76 min, 95% CI 12.41–53.11 min, *P* = 0.002) (Additional file 1: Figure S1). There were no significant differences between the two groups in terms of operative bleeding (WMD 66.99 ml, 95% CI − 33.58–167.56 ml, *P* = 0.192) (Additional file 1: Figure S2) and hospital stay (WMD − 0.15 days, 95% CI − 1.14–0.84 days, *P* = 0.766) (Additional file 1: Figure S3).

**Fig. 6:**
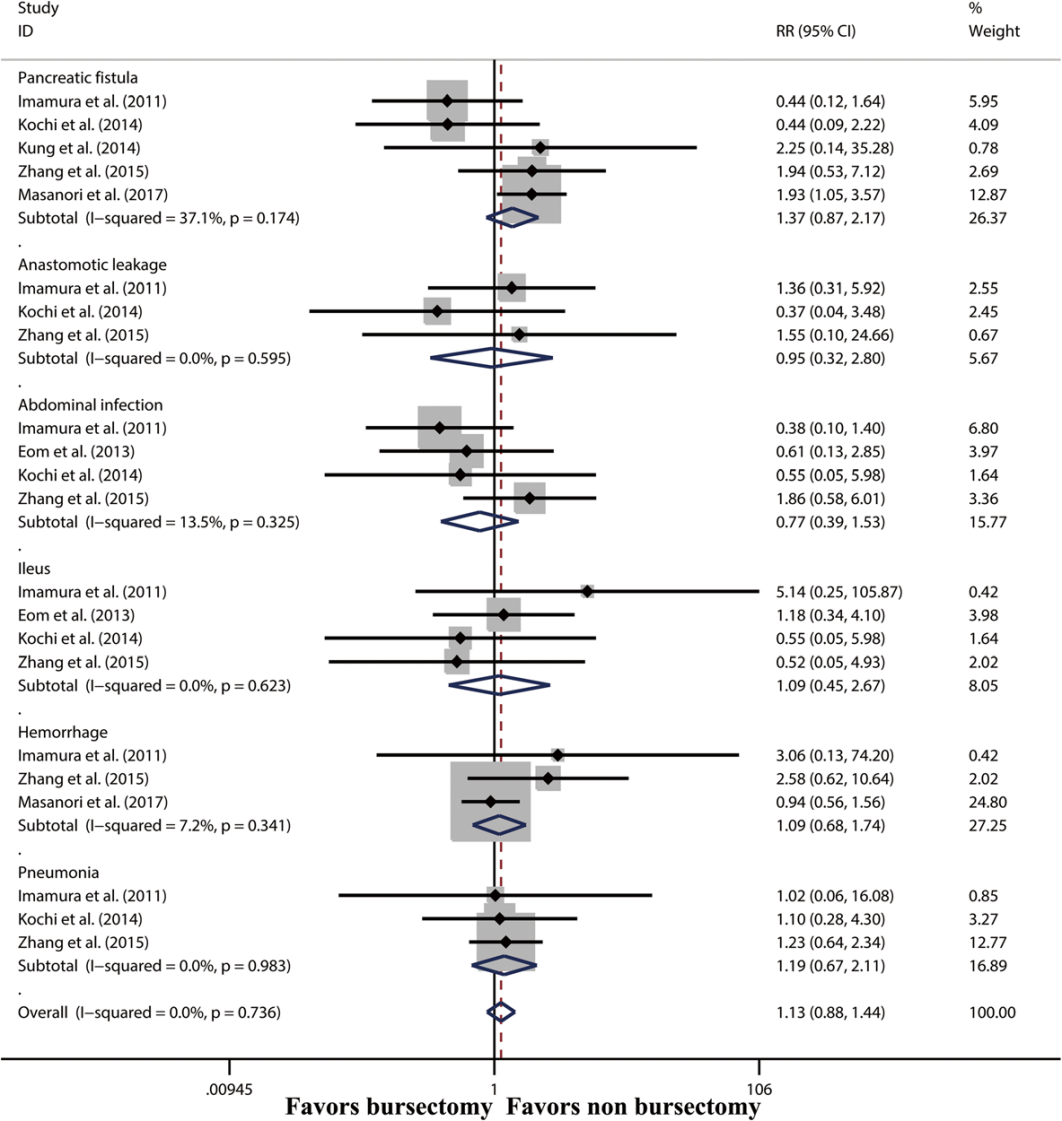
Forest plot and meta-analysis of pancreatic fistula, abdominal infection, ileus, haemorrhage and pneumonia

Five studies [7, 9, 17, 18, 20] compared the overall postoperative complication between the bursectomy group and non-bursectomy group. No significant difference was found between the two groups (RR 1.08, 95% CI 0.90–1.28, *P* = 0.421) (Additional file 1: Figure S4). Subgroup analysis showed that there were no significant differences between the two groups in terms of pancreatic fistula, abdominal infection, ileus, haemorrhage and pneumonia (Fig. 6).

**Fig. 7:**
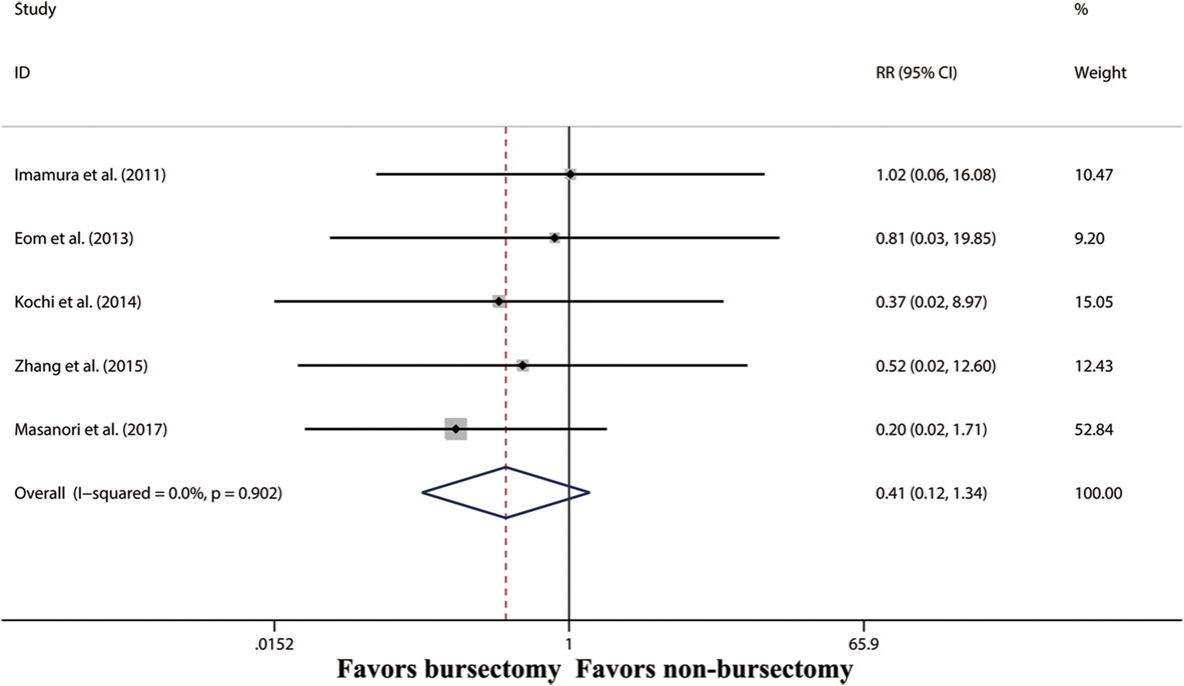
Forest plot and meta-analysis of mortality

The pooled RR for overall postoperative mortality between the two groups was 0.44 (95% CI 0.13–1.53, *P* = 0.195) (Fig. 7).

### Publication bias

Begg’s funnel plot indicated that there was no obvious publication bias in OS between the studies (*P* = 0.255) (Additional file 1: Figure S5).

## Discussion

The operative advantages such as the aesthetic plane of dissection appealed several experienced surgeons to perform bursectomy. However, whether AGC patients can benefit from bursectomy remained uncertain. The pooled results of the present meta-analysis showed that AGC patients cannot benefit from bursectomy with regard to oncological outcomes.

As recommended by the UICC/AJCC TNM classification, excision of a minimum of 15 LNs is recommended for gastric cancer in order to allow reliable staging. Theoretically, complete bursectomy is possible to help harvest more subpyloric LNs. Eom et al. reported that the bursectomy group had a higher number of harvested LNs (57.6 vs. 48.5, *P* < 0.001) [17]. However, other studies reported inconsistent results [7, 18, 20]. The pooled results of the present meta-analysis revealed that the number of dissected LNs was not higher in the bursectomy group. In addition, this meta-analysis suggested that bursectomy was a time-consuming procedure with a longer operation time.

Additional surgical procedures correspond to a higher potential risk of postoperative complications and mortality. Complete bursectomy requires the complete dissection of the pancreatic capsule, which has the possibility of increasing the incidence of pancreatic fistulas and intestinal obstruction after bursectomy. The present meta-analysis demonstrated that the complications, including pancreatic fistula, abdominal infection, ileus, haemorrhage and pneumonia, was similar between the bursectomy group and non-bursectomy group, indicating that bursectomy was safe. However, it is important to note that the majority of the included studies were from high-volume institutions where D2 gastrectomy with bursectomy was performed by experienced surgeons. The data from a Dutch trial demonstrated that D2 lymphadenectomy in the low-volume centres was associated with higher postoperative complications and mortality [3]. Moreover, bursectomy is a complicated and technique-dependent procedure. Therefore, we believe that bursectomy can be safely performed mostly by experienced surgeons.

Oncologic benefit is necessary for bursectomy to be recommended as a mandatory procedure in D2 gastrectomy for AGC. A retrospective study reported that there was no significant difference in OS for AGC, including tumours invading the serosa of the posterior gastric wall [17]. Kochi et al. also reported comparable OS between the bursectomy group and non-bursectomy group (5-year OS, 85.8 vs. 80.8%, *P* = 0.60) [18]. However, the long-term outcomes of a multicentre RCT demonstrated that after adjusting for possible confounding covariates, bursectomy was associated with favourable OS [8]. At the 2017 ASCO-GI meeting, Masanori et al. reported the primary results of JCOG1001, indicating that the 3-year OS was similar between the bursectomy group and non-bursectomy group [9]. Pooling the results from previous studies, this meta-analysis showed that bursectomy was not associated with DFS and OS. Moreover, gastric cancer patients with serosa-penetrating tumours may not benefit from bursectomy in terms of changes in OS.

Several factors could account for the failure of bursectomy to confer survival benefit in AGC. First, the omental bursa is not a closed cavity, and it opens to the abdominal cavity through Winslow’s foramen. By comparing carcinoembryonic antigen (CEA) or mRNA in the omental bursa and two other sites of the abdominal cavity, Yamamura et al. demonstrated that viable cancer cells in the omental bursa can migrate into other abdominal cavity [21]. Second, it is possible that adjuvant chemotherapy is a reason for the lack of survival benefit of bursectomy. Adjuvant chemotherapy has been confirmed to improve OS and DFS in gastric cancer patients after D2 gastrectomy [4, 5]. Masanori et al. reported that AGC patients could no longer benefit from bursectomy if they received adjuvant chemotherapy after D2 gastrectomy [9]. Therefore, we believe that the survival benefit of bursectomy for AGC may be limited in the era of adjuvant chemotherapy.

There were several limitations in this meta-analysis. First, literature search was limited to English language. Secondly, four included studies were retrospective, and one study was an abstract. Thirdly, the majority of included studies were from eastern Asia in high-volume centres, which limits the application of the results of this meta-analysis. Finally, several analyses showed significant heterogeneity.

## Conclusions

In summary, this meta-analysis indicated that bursectomy is a time-consuming procedure without increasing the number of harvested LNs. Although bursectomy can be safely performed by experienced surgeons without increasing complications and mortality, it cannot prolong OS and DFS of AGC patients, including patients with serosa-penetrating tumours. Therefore, bursectomy should not be recommended as a standard procedure for AGC.

## Additional file

Additional file 1: Supplementary materials. (DOCX 307 kb)
