# Supplementary material for: Bursectomy for advanced gastric cancer: an update meta-analysis
Source: World J Surg Oncol. 2018 Mar 27;16:66. doi: 10.1186/s12957-018-1354-1 (PMC5872552; doi:10.1186/s12957-018-1354-1)
Supplement: Supplementary file 1 — Supplementary materials. (DOCX 307 kb) [file 12957_2018_1354_MOESM1_ESM.docx]

**
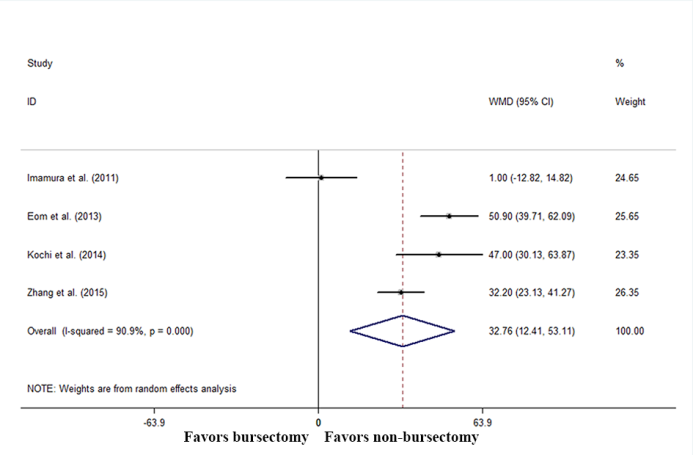
Fig. S1** Forest plot demonstrating the pooled difference between bursectomy group and non-bursectomy group regarding operation time.

**
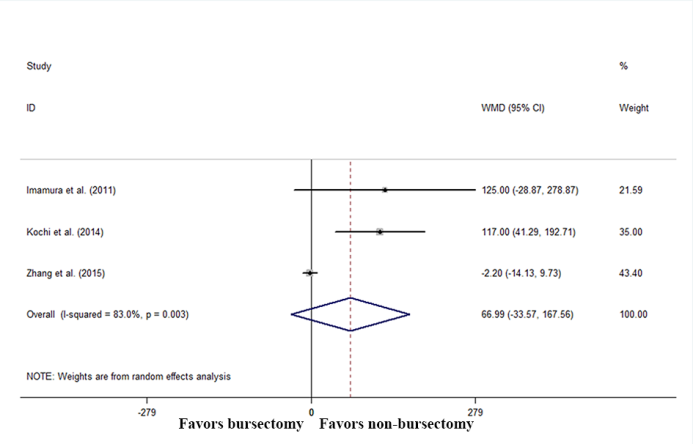
Fig. S2** Forest plot demonstrating the pooled difference between bursectomy group and non-bursectomy group regarding operative bleeding.

**
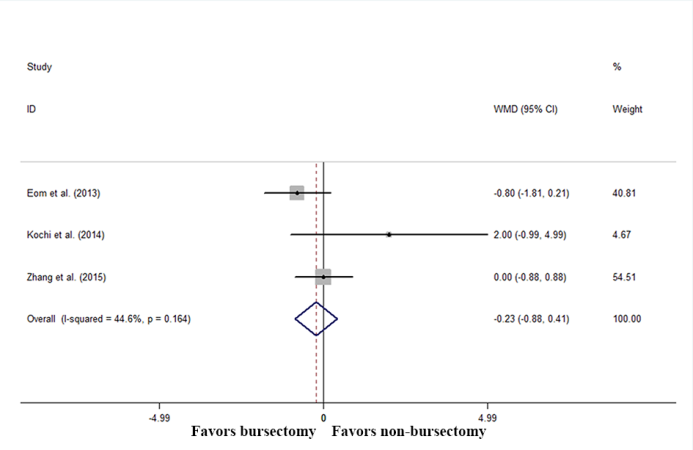
Fig. S3** Forest plot demonstrating the pooled difference between bursectomy group and non-bursectomy group regarding hospital stay.

**
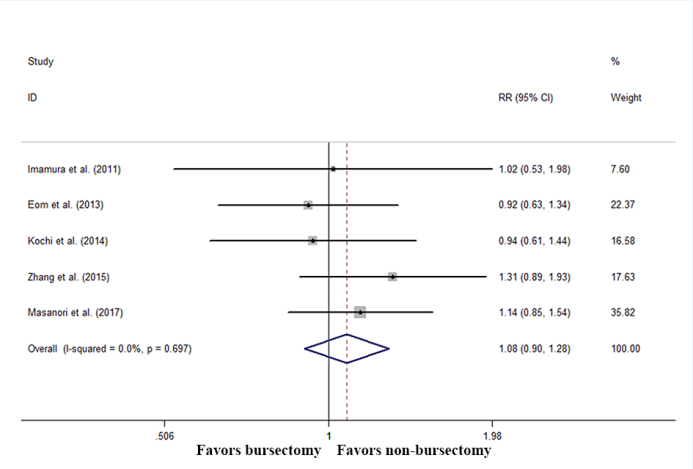
Fig. S4** Forest plot demonstrating the pooled difference between bursectomy group and non-bursectomy group regarding postoperative complication.

**
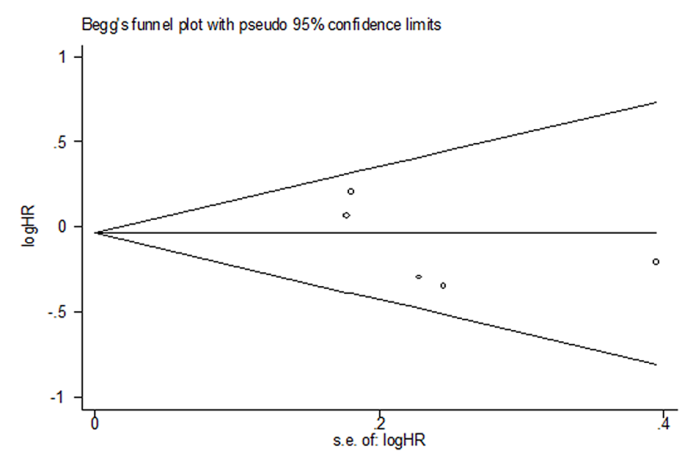
Fig. S5** Begg’s funnel plot illustrating publication bias in OS between bursectomy group and non-bursectomy group.
